# Measuring the health impact of human rights violations related to Australian asylum policies and practices: a mixed methods study

**DOI:** 10.1186/1472-698X-9-1

**Published:** 2009-02-03

**Authors:** Vanessa Johnston, Pascale Allotey, Kim Mulholland, Milica Markovic

**Affiliations:** 1 Menzies School of Health Research and Institute of Advanced Studies, Charles Darwin University, PO Box 41096, Casuarina, NT 0811, Australia; 2 Centre for Public Health Research, School of Health Sciences and Social Care, Brunel University, West London, Uxbridge, UB8 3PH, UK; 3 Infectious Disease Epidemiology Unit, London School of Hygiene and Tropical Medicine, Keppel Street, London, WC1E 7HT, UK; 4 School of Psychology, Psychiatry and Psychological Medicine, Monash University, Clayton, Victoria, Australia

## Abstract

**Background:**

Human rights violations have adverse consequences for health. However, to date, there remains little empirical evidence documenting this association, beyond the obvious physical and psychological effects of torture. The primary aim of this study was to investigate whether Australian asylum policies and practices, which arguably violate human rights, are associated with adverse health outcomes.

**Methods:**

We designed a mixed methods study to address the study aim. A cross-sectional survey was conducted with 71 Iraqi Temporary Protection Visa (TPV) refugees and 60 Iraqi Permanent Humanitarian Visa (PHV) refugees, residing in Melbourne, Australia. Prior to a recent policy amendment, TPV refugees were only given temporary residency status and had restricted access to a range of government funded benefits and services that permanent refugees are automatically entitled to. The quantitative results were triangulated with semi-structured interviews with TPV refugees and service providers. The main outcome measures were self-reported physical and psychological health. Standardised self-report instruments, validated in an Arabic population, were used to measure health and wellbeing outcomes.

**Results:**

Forty-six percent of TPV refugees compared with 25% of PHV refugees reported symptoms consistent with a diagnosis of clinical depression (p = 0.003). After controlling for the effects of age, gender and marital status, TPV status made a statistically significant contribution to psychological distress (B = 0.5, 95% CI 0.3 to 0.71, p ≤ 0.001) amongst Iraqi refugees. Qualitative data revealed that TPV refugees generally felt socially isolated and lacking in control over their life circumstances, because of their experiences in detention and on a temporary visa. This sense of powerlessness and, for some, an implicit awareness they were being denied basic human rights, culminated in a strong sense of injustice.

**Conclusion:**

Government asylum policies and practices violating human rights norms are associated with demonstrable psychological health impacts. This link between policy, rights violations and health outcomes offers a framework for addressing the impact of socio-political structures on health.

## Background

Perhaps most notable in the evolution of the health and human rights paradigm is the recognition that many human rights violations (HRVs) have adverse consequences for health. The health effects of blatant violations are obvious, such as those arising from torture [1]. Additionally, there are more subtle but nonetheless important right violations that may impact on health, such as the absence of basic health care systems or tolerance of discrimination against minority groups [2].

While the link between HRVs and health seems intuitive, beyond the obvious health effects of torture [3,4], the empirical assessment of the health impacts of HRVs is in its infancy [5]. Put simply, we are yet to understand fully the nature of these relationships, how they interact and their importance for public health practice [6]. More research is required to assess the broad health impact of HRVs and to identify the pathways by which violations become embodied as poor health outcomes.

Over recent years, the asylum policies of successive Australian governments have generated much controversy; namely the mandatory detention of undocumented asylum seekers while their refugee claims are processed, and the introduction of a time limited (3–5 years) Temporary Protection Visa (TPV) to asylum seekers despite the verification of their claim for refugee status under the 1951 UN Refugee Convention. The temporary protection regime was introduced in 1999 and until very recently was a core component of Australia's policy approach to asylum seekers. However, in 2008, a newly elected Labour government announced it would abolish the temporary visa scheme and would revert to issuing permanent protection status to bonefide refugees. Additionally, under new detention arrangements, asylum seekers will be detained only as a last resort (e.g. if they are shown to pose a risk to the community) and for the least practicable time [7].

Under the TPV policy refugees were excluded from or had limited access to a range of federal government funded benefits and services such as full income support benefits, free English language tuition, government supported university places, interpreting and settlement services [8]. Additionally, TPV refugees were barred from family reunion programs. By contrast, individuals entering via Australia's offshore humanitarian program have their refugee status established and permanent humanitarian visa (PHV) issued prior to arrival in Australia. This entitles them to the full range of services afforded to permanent residents.

Australia has ratified several human rights treaties, including the *International Covenant on Civil and Political Rights (ICCPR)*, the *International Covenant on Economic, Social and Cultural Rights (ICESCR)*, the *1951 Convention Relating to the Status of Refugees *and the *Convention on the Rights of the Child*. The Australia government, as a party to these treaties, is obliged under customary international law to protect the human rights of all persons within its territory and subject to its jurisdiction [9]. The detention and temporary protection of asylum seekers, as practiced in Australia, are widely believed to have violated human rights norms under international law [10,11] (see Table 1 for a summary of the key policies and practices that have received the most scrutiny in relation to their impact on human rights [11-17]).

**Table 1:** Human rights implications of Australian asylum policies and practices

Australian asylum policies and practices	Human rights implications
Detention of asylum seekers	Arbitrary detention of asylum seekers is prohibited by Article 9(1) of the ICCPR. For the lawful detention of asylum seekers to be considered non-arbitrary, it should only be administered for the shortest time possible and be a reasonable, necessary and proportionate means to achieving a legitimate outcome, while giving due regard to alternative means which are less imposing on an individuals' rights [12]. Australia's history of automatic, mandatory detention of asylum seekers until their claims are finalised may be categorised as arbitrary in practice (in almost all cases, except those detained for a brief period), as the deprivation of liberty for an indeterminate period is difficult to justify on the grounds that it is a reasonable means of achieving a legitimate aim (i.e. for the purpose of granting a visa or removal from the territory) [13].

Temporary protection of refugees	Under the Refugee Convention, a signatory State is not required to provide permanent residence to refugees to meet its Convention obligations. However, temporary protection, as outlined by the United Nation High Commissioner for Refugees (UNHCR) should in general, only be applied to large groups of asylum seekers, who come en masse into a receiving country and threaten to overwhelm the administrative capacity of that country [14]. By applying a blanket temporary protection policy to all undocumented asylum seekers, it is arguable that Australia was "overly restrictive in its interpretation and application of this key element of the Convention." [15, para. 4] As such, the legitimacy of the policy under international law is questionable, as it is contrary both to the spirit of the Refugee Convention as well as accepted international standards [15,16].

Restricted entitlements of TPV refugees	Article 34 of the Refugee Convention requires host States 'as far as possible' to 'facilitate the assimilation and naturalization of refugees'. By denying TPV holders certain social and welfare rights, the Australian policy dismissed those sections of the Refugee Convention, which are aimed towards assisting refugees, an already vulnerable group, to return to a "situation of national protection in a new country, if not their own, as soon as possible" [11]. To assist in this process, the Refugee Convention affords refugees "the most favorable treatment" accorded foreign nationals in the resettlement country, with respect to employment (Refugee Convention, 1951, Article 17). It is arguable that the restrictions on TPV refugees in accessing services to assist them with employment opportunities were not in keeping with this obligation. In relation to public relief (Article 23) and social security (Article 24), refugees are to be given equal access as nationals [17], by stark contrast to the restricted welfare entitlements offered to TPV refugees in Australia. Finally, the Australian temporary protection regime was not consistent with UNHCR policy and the practice of other States (e.g. in the European Union) whereby full Refugee Convention rights should be granted if return remains impossible after a few years [11].

Two previous cross-sectional studies have investigated the impact of asylum policies on the health of refugees in Sydney, Australia. These studies reported that both negative detention experiences (e.g. prolonged detention or stressful events whilst in detention, such as exposure to violence) and temporary protection were associated with adverse mental health outcomes for refugees subjected to this regime [18,19]. Separate qualitative research has highlighted that restricted access to settlement services combined with the persistent uncertainty about their residency status have impacted negatively on the integration of TPV holders into the community [20]. In particular, psychosocial stressors, such as social isolation, a lack of control over life circumstances and a collective sense of injustice appear to be salient in the experience of TPV holders [20,21].

Drawing from this earlier research, we aimed to further explore the relationship between health and human rights in this specific context and investigate the mediating pathways which link the two. Our underlying premise was that the Australian asylum policies and practices were a violation of human rights. To address the study aim, we designed the first mixed methods study with this population. Specifically, we posed two research questions:

Are Australian asylum policies and practice of detention and temporary protection associated with adverse health outcomes?

Are psychosocial stressors experienced by TPV refugees associated with adverse health outcomes amongst this group?

## Methods

### Participants

Data were collected between November 2004 and October 2005. The study group comprised of adult (≥ 18 years) TPV and PHV Iraqi refugees who arrived in 1999 (when the TPV was introduced) or later and were living in four adjacent local geographical areas in the northern suburbs of Melbourne, Australia. The study site was chosen because of the concentration of newly arrived Iraqi refugees in these areas [22]. Participants were excluded if they could not speak Arabic or English, did not hold a humanitarian visa, and/or had been living in the community (i.e. outside of detention) for less than six months.

The study used quantitative and qualitative methods. A cross sectional survey included both TPV and PHV refugees. The qualitative arm involved semi-structured interviews but was restricted to TPV refugees and a purposive sample of local service providers.

### Sampling

The lack of a community sampling frame (i.e. TPV holders were not represented on any publicly accessibly database) constrained the sampling strategy for the survey. Based on community profiles from both the Department of Immigration and Citizenship (DIAC) settlement database (which collects data on PHV refugees) and local service providers, which have informally gathered data on TPV refugees [23], non-probalistic sampling techniques described by De Vaus [24] were used to purposively select participants who characterised the diversity of the Iraqi refugee population in Melbourne. Thus, recruitment targeted women and men of varying ages, educational backgrounds and family compositions (e.g. intact and non-intact nuclear families). It was not possible to systematically match TPV and PHV participants on demographics such as sex or age because of the "hard to reach" nature of refugee populations [25]. Participants were recruited through community organisations such as Migrant Resource Centres and non-government organisations providing services to refugees in the study site. Community health centres were not included as points of contact in order to avoid over-representation of 'patients'. Refugees who did not utilise these community services were accessed by snowballing within established community networks.

A sample size estimate was calculated based on prevalence data from then preliminary results of a community-based study with Iraqi refugees in Sydney. The provisional findings from this study found a prevalence rate of self-reported depression of approximately 70% in the TPV group and 40% in the PHV group (pers. comm., Dr Steel [University New South Wales], 24 May 2004). Taking a conservative estimate based on this prevalence data, we calculated that 60 subjects per group would have 80% power to achieve a statistically significant result at a 2-sided 5% level, if the true proportions were 55% and 25% for the TPV and PHV groups respectively [26].

The survey sample provided a sampling frame for the nested interviews that were conducted with 16 TPV holders. Using theoretical sampling, we drew on previous research, that suggested that age, gender, education and marital status influence post-migration mental health [27,28] and sampled to reflect the diversity across these factors. The grounded theory approach influenced sampling, data collection and analysis for these in-depth interviews [29].

### Outcome measures

The questionnaire for the survey was designed to collect basic sociodemographic data and to assess socioeconomic and psychosocial stressors and general health outcomes. Socioeconomic stressors were defined as obstacles in accessing core social determinants of health, such as employment and training, language tuition, housing, health and welfare services and relate directly to the entitlements of migrants under international human rights and refugee law. The standardized instruments used to assess the three psychosocial constructs tested were: the *Medical Outcomes Study Social Support Scale *(MOS-SS) [30], the *Perceived Constraints *subscale of the Lachman and Weaver [31]*Sense of Control Scale*, and the *State-Trait Anger Expression Inventory *(*STAXI*) [32], respectively. The STAXI scale was chosen as a 'proxy' measure of injustice because empirical research has demonstrated that anger is the most consistent emotional reaction elicited by perceived injustice within and between cultures [33,34]. We posed an open ended question to assess the experience of personal and family trauma. The decision to avoid more established scales such as the Harvard Trauma Questionnaire was based on ethical concerns that administration of the questionnaire may cause distress in the context of possible repatriation of TPV refugees back to Iraq (i.e. at the expiry of their temporary visa).

Health outcomes were measured for self-reported physical health (*SF-36 General Health Scale *[35]; *SF-36 Physical Functioning Scale*[35]), psychological health (*Hopkins Symptom Checklist-25; HSCL-25 *[36]) and personal wellbeing (*Personal Wellbeing Index; PWBI *[37]). All self-rated health measures had previously been validated in an Arabic population. The survey was presented in English and in Arabic and was self-administered in the presence of the first author (VJ) and a bilingual, bicultural project worker. We applied Brislin's [38] translation and back-translation method using two bilingual professionals; the original and back-translated versions were assessed for meaning equivalence. The questionnaire was pilot tested, which after some amendments (e.g. reconciling some minor semantic differences), demonstrated good face validity.

Semi-structured interviews with refugees aimed to explore in greater depth the perceived socioeconomic and psychosocial stressors associated with Australian migration policies and the impact of these on their health and wellbeing.

### Data analysis

Preliminary univariate analyses were performed to test for differences across the two visa groups in relation to sociodemographic characteristics, the experience of socioeconomic and psychosocial stressors, and self-rated health outcomes. The proportion of the sample that reported symptoms consistent with a diagnosis of clinical depression on the HSCL-25 scale was calculated, according to an established DSM-IV derived algorithm [39]. The HSCL-25 scale was also used as a continuous measure in multivariate analyses. A Personal Wellbeing (PWB) score was calculated as the average of the items, converted into units of Percentage Scale Maximum (%SM) (using the formula: mean PWB score/10 × 100) [40].

Where normal probability plots of continuous data indicated a normal distribution, we used parametric statistical tests in our analyses. Nonparametric measures of association were used to ascertain group differences across categorical variables or where normality assumptions of continuous variables could not be strictly met. Where possible, data that were skewed were log transformed to achieve a normal distribution to enable analyses using t-tests and regression [41].

Standard linear regression models were used to asses the impact of visa status on selected self-rated mental health outcomes, after controlling for potential confounding factors. To investigate the association of independent variables with self-rated health outcomes amongst TPV holders, variables were simultaneously entered into a linear regression model for participants in this visa category. Analyses were undertaken with Statistical Packages for Social Sciences (SPSS) for Windows.

The first author (VJ) employed thematic coding to explore the qualitative data. This involved identifying themes and sub themes in the data, refining and reducing these initial themes, building hierarchies and linking themes into a broader theoretical framework [42]. Pseudonyms were used in the reporting of verbatim quotations from participants. Qualitative data were managed using the computer software program, Atlas-ti (Version 5).

### Ethics

The study received ethics approval from the University of Melbourne Human Research Committee.

## Results

### Survey Results

Seventy-one TPV refugees and 60 PHV refugees completed questionnaires. Table 2 presents the key sociodemographic characteristics of participants in the two different visa categories. These characteristics were broadly representative of the wider Iraqi refugee population in the study site according to available data from the DIAC and service providers located in the area. These sources indicated that the average age for Iraqi refugees is mid-adulthood, the majority are married, Arabic and generally well educated [23].

**Table 2:** Summary of key sociodemographic characteristics of study sample by visa status

Characteristic	TPV refugees n = 71	PHV refugees n = 60	p-value
Age, years mean, (SD)	35.1 (11.0)*	34.5 (12.2)	0.76
Female	31 (44)	33 (55)	0.26
Married/de facto	39 (55)	36 (61)	0.60
Ethnicity – Arabic	59 (83)	49 (82)	0.73
Completed secondary education	50 (73)	45 (76)	0.77
Personal and/or family experience of persecution	37 (52)	23 (38)	0.16
Time in Australian community, months mean, (SD)	42.6 (8.6)	38.7 (20.4)	0.14
Separation from spouse and/or child in Australia	11/37^† ^(30)	5/36 (14)	0.18
Received welfare payments in the last 6 months	53 (73)	54 (90)	0.04

* Number of participants with positive responses, % in parentheses

^† ^Denominator is number of participants with spouse and/or children

There was no statistically significant difference between TPV and PHV Iraqi refugees with regard to their sociodemographic and cultural backgrounds and in broad terms, had similar pre-arrival refugee experiences. Where the two groups differed substantially was in their post arrival experiences and entitlements under Australia's migration legislation.

### Experience of socioeconomic and psychosocial stressors

The effects of the asylum policies were reflected in the socioeconomic stressors reported by refugees. A significantly greater proportion of TPV refugees reported obstacles with 'access' to services and other settlement-related resources compared with the PHV group, across all service categories except for health, where the proportions were similar in the two groups (see Table 3). The main reason that TPV refugees gave for the problems they had in accessing tertiary education, English classes and welfare, was their limited entitlements to these services (Results not shown), which was a core part of the TPV policy.

**Table 3:** Comparison of socioeconomic stressors by visa status

Socioeconomic stressor	TPV refugees n (%)	PHV refugees n (%)	p-value
Obstacles accessing accommodation	41/71* (58)	22/60 (37)	0.03
Obstacles accessing education/job training	43/68 (63)	11/52 (21)	< 0.001
Obstacles finding employment	40/61 (66)	8/34 (24)	< 0.001
Obstacles accessing English language tuition	39/67 (58)	8/59 (14)	< 0.001
Obstacles accessing health services	24/70 (34)	21/56 (38)	0.85
Obstacles accessing welfare services	18/68 (27)	2/58 (3)	0.001
Number of obstacles to 'access' reported – 3 or more^†^	42/70 (60)	10/58 (17)	< 0.001
At any time under current visa been unable to afford:			
Food	10/71 (14)	2/60 (3)	0.07
Clothing	16/71 (23)	9/60 (15)	0.38
Accommodation	24/71 (34)	8/60 (13)	0.01
Medications	16/71 (23)	2/60 (3)	0.003
Number of essential items (food, clothing, accommodation, medication) unable to afford – 1 or more	28/71 (39)	13/60 (22)	0.05

* The data in this table include only those refugees who had attempted to access these services as the denominator

^† ^Questions related to 'access' to six services/settlement-related resources: accommodation, education/job training, employment, English language tuition health and welfare services.

The results also indicated a significant difference between the two visa categories in their experience of psychosocial stressors, with TPV refugees reporting, on average, less social support, more life constraints and higher state anger scores (see Table 4).

**Table 4:** Comparison of psychosocial stressors by visa status

	TPV refugees (n = 70)	PHV refugees (n = 60)	p-value
Medical Outcomes Study Social Support (MOS-SS) score [0–100 scale] (median)	28.1	45.3	0.01
Perceived constraints (PC) score [1–7 scale] mean, (SD)	4.6 (1.3)	4.0 (1.2)	0.003
STAXI score			
State-Anger score [10–40 scale] (median)	18.5	11.5	≤ 0.001
Trait-Anger score [10–40 scale] mean, (SD)	21.8 (7.1)	20.7 (5.2)	0.3
STAXI State/Trait Anger Ratio (geometric mean)*	0.90	0.64	≤ 0.001

* Original STAXI State/Trait Anger Ratio scale was analyzed in logarithmic scale

### Self-rated health outcomes

While there was no significant difference between the two groups in the assessment of their 'general health' (GH) and their 'physical functioning' (PF), there was a highly significant difference between the groups in their reporting of psychological distress and their wellbeing (see Table 5).

**Table 5:** Comparison of self-rated health outcomes by visa status

	TPV refugees (n = 70)	PHV refugees (n = 60)	p-value
SF-36 General Health (GH) score mean, (SD)	55.5 (26.5)	59.0 (21.9)	0.41
SF-36 Physical Functioning (PF) score mean, (SD)	70.5 (30.4)	72.0 (28.5)	0.77
Psychological distress (HSCL-25) mean, (SD)	2.1 (0.7)	1.6 (0.5)	< 0.001
Depression (HSCL-25) %	46	25	0.003
Personal Wellbeing Index (PWBI) % SM mean, (SD)	53.2 (22.2)	67.0 (17.2)	< 0.001

Mean psychological distress scores were higher among TPV refugees compared with PHV refugees. Forty-six percent (46%) compared with 25% of permanent refugees reported symptoms consistent with a diagnosis of clinical depression. Self-rated personal wellbeing was lower among TPV refugees (mean PWBI of 53% SM), compared with PHV refugees (67% SM).

### Contribution of visa to psychological distress

Standard linear regression modelling was used to assess the contribution of visa category to the HSCL-25 scores, when the variance explained by other relevant variables was controlled for (see Table 6). The additional variables included in the regression equation were age, gender, and marital status; factors previously identified as predictors of psychological health amongst resettling refugees. A history of persecution was originally included in the model but contributed little to the variance in HSCL-25. When it was removed from the model, there was no effect on the regression coefficients for all of the other included variables, indicating that it was a redundant variable in the model. Previous research has found an association between exposure to trauma and psychological health amongst refugees [3,4]. In the present study the proportions of participants in both groups reporting persecution were not significantly different, so there is no reason to suspect that pre-migration trauma played an important role in explaining differences in health outcomes between the temporary and permanent refugee groups.

**Table 6:** Standard linear regression model assessing predictors of psychological distress (HSCL-25) (n = 130)

Variable	B*	95% Confidence interval	p-value
Visa			
TPV (cf PHV)	0.50	0.30, 0.71	≤ 0.001
Gender			
Female (cf Male)	0.21	-0.01, 0.42	0.06
Marital Status			
Never married (cf Divorced/Widowed)	-0.72	-1.19, -0.25	0.003
Married (cf Divorced/Widowed)	-0.63	-1.04, -0.24	0.003
Age (for 10 years difference)	0.04	-0.07, 0.15	0.43

*Unstandardised regression coefficients (B) are provided with 95% confidence intervals.

The regression model explained an estimated 27.4% of the variance in HSCL-25 scores (R squared = 0.274) amongst Iraqi refugees [F (5,123) = 9.22, p = ≤ 0.001]. Visa and marital status made a significant independent contribution to variation in HSCL-25 scores.

### Determinants of psychological health amongst TPV holders

Initially, the relationship between self-rated psychological distress (HSCL-25) of TPV refugees, and 12 independent variables was assessed. The range of variables chosen was based on prior research that had suggested a relationship with mental health outcomes or was hypothesised in this study to show a correlation. These were: age, gender, education, marital status, history of persecution, months in detention, separation from spouse and/or child in Australia, number of difficulties accessing services, number of essential items of daily living unable to afford, perceived life constraints, social support and anger.

A regression model was fitted to assess the contribution of five independent variables that demonstrated significant univariate associations. These were perceived life constraints, social support, anger, gender, marital status and 'number of daily essential items' refugees were unable to afford since arriving in Australia (see Table 7 for correlation matrix). The results of analysis of the regression model are presented in Table 8.

**Table 7:** Correlations among variables in regression model to assess determinants of psychological distress (HSCL25) amongst TPV holders

	HSCL25	Gender	No. essential items unable to afford	MOS-SS score	PLC score
**Gender**	0.34 (0.002)*				
**No. essential items unable to afford**	0.37 (0.001)	0.16 (0.09)			
**MOS-SS score**^†^	-0.41(< 0.001)	0.21 (0.04)	-0.20 (0.05)		
**PC score **^‡^	0.39 (< 0.001)	0.19 (0.06)	0.32 (0.004)	-0.25 (0.02)	
**STAXI S/T Anger Ratio **^§^	0.41 (< 0.001)	0.02 (0.43)	0.19 (0.06)	-0.28 (0.01)	0.34 (0.02)

* p values in parentheses

^† ^Medial Outcomes Study Social Support (MOS-SS) score

^‡ ^Perceived Constraints (PC) score

^§ ^STAXI State/Trait Anger Ratio. Original state/trait ratio scale has undergone logarithmic transformation

**Table 8:** Standard linear regression model to assess determinants of psychological distress (HSCL-25) amongst TPV refugees (n = 70)

Variable	B	95% Confidence interval	p-value
Gender			
Female (cf Male)	0.50	0.18, 0.81	0.002
No. essential items unable to afford			
One or more (cf Zero)	0.24	-0.06, 0.53	0.11
Marital Status			
Divorced/widowed (cf Never married)	0.27	-0.29, 0.84	0.34
Married (cf Never married)	-0.05	-0.35, 0.25	0.73
MOS-SS score (for 20 points difference)	-0.16	-0.24, -0.06	0.003
PC score	0.05	-0.07, 0.17	0.42
STAXI S/T Anger Ratio (for 0.1 point difference)	0.15	0.03, 0.28	0.02

This regression model explained an estimated 42.8% of the variance in HSCL-25 scores (adjusted R squared = 0.428) amongst Iraqi TPV refugees [F (7, 60) = 8.17, p ≤ 0.001]. Three variables made a statistically significant contribution to HSCL-25 (psychological distress) scores. They were gender, social support and anger.

### Qualitative Results

The quantitative data highlighted the specific psychosocial variables (e.g. social support) that were associated with adverse mental health outcomes. However, the qualitative data were critical in exploring the antecedents of these stressors, and how they impacted on health. Interviews with TPV refugees revealed that they felt socially isolated owing to policy restrictions on reuniting with family members, and the structural barriers that prevented them from fully participating in society (e.g. not being able to access government-funded English tuition). While their mainstay of support were other TPV refugees, most admitted that giving and getting support from other TPV holders could be emotionally draining and a burden some wished to avoid at various times. This was particularly evident during the period when Iraqi TPV refugees were applying for permanent protection and many felt additional anxiety about the outcome of their claims:

Q: During this difficult time in your life, who can you turn to for some support for you?

A: Who am I going to talk with? I cannot talk to anyone. Everyone is feeling hopeless because eighty percent of Iraqis they being refused [permanent protection]. It is hard to get support from people who have all these problems. It is hard to talk to other TPV holders...If there were some percentage of people that are accepted and they are happy, then they can support the smaller percentage of people who are struggling. But now – the situation – most of the people they are in a bad situation and so they cannot support each other. (Kadar – male, aged 43)

Additionally, some interviewees were particularly sensitive to not wanting to overburden important social ties. This was particularly the case with women, many of whom expressed the desire not to contribute additional stress to their children and husbands:

...I am the woman who supports everybody else and I can't speak to anybody about my feelings. I have to keep it inside. I can't talk. I cannot speak my feelings to my children, because they get upset. I have to keep my feelings because of the children. (Alima – female, aged 48)

In part, women's predilection to keep their 'worries' to themselves stems from their gendered roles, whereby Iraqi women are largely responsible for the rearing of children and are traditionally seen as the keepers of harmony in the home [43]. Consistent with this, the qualitative interviews revealed that women generally viewed their migration experiences through the lens of the family, with particular reference to their children for whom they felt ultimately responsible. Equally, when they talked about the future, the burden of anxiety was layered with not only fears for themselves, but also fears for their children:

For me, I want the permanent visa not to travel or go to another place but just to make sure that my children have a safe place for their future. My son he forgot Arabic, now he speaks like a baby in Arabic...He likes the school here and the teachers and his friends. How can I take him back to Iraq at this time? I always worry so much for my children. I am driving my family crazy with my worry. (Falak – female, aged 25)

Men on the other hand, generally presented themselves more autonomously. The family context was not a major theme in their migration narratives; rather loss of personal status and professional identity were more the norm:

Maybe if I got permanent visa, I will start my life here again. Maybe I can make my own job, my own business in the future and I can take a place here in Australia I think I still have the time to do something for Australia if I get the permanent visa. (Husam – male, aged 45)

The qualitative data also revealed that TPV holders perceived that they had little agency over their lives and they expressed anger at the lack of control over the refugee determination process, be that in detention or later when applying for a permanent protection visa. Their anger and frustration were compounded when after gaining refugee status, the policies in place imposed further barriers to achieving otherwise 'normal' resettlement goals. Family reunion, access to language facilities, education and employment remained out of their reach:

You feel like a person moving around in circles. I can't take a step forward, I can't help myself, and I can't help with this community. (Mahir – male, aged 40)

It's hard because of TPV. Makes you feel frustrated and you can't develop yourself. You feel insecure and so you stay like frozen. (Emir – male, aged 43)

Perception of injustice was another key precipitant of anger. Interestingly, one of the more common reasons the participants gave as to why they felt the policies were unjust related to human rights. Although only one specified an international treaty (the Refugee Convention) as a source of rights that *should *be afforded refugees, several outlined specific rights that are contained in binding international law, and which they were being denied as a result of the Australia's policies and practices. As highlighted in the following excerpt, these included the right to not be detained arbitrarily and the right to religious freedoms (Article 9 and 18 respectively of the ICCPR):

Q: What are your feelings about this policy of detention?

A: This is such an unfair policy, especially for children. They come here looking for a safe place; they are not criminals. We are just normal people looking for a safe place...There are no human rights coming to us here...

Q: What human rights do you think you were missing in [the detention centre]?

A: They treat the women disrespectfully. [The women] are desperate and nobody listen to them. You know our tradition, we are Muslim and the women can't go to male doctors, she needs a female and we can't find this is in [detention]...We are all people looking just for freedom but when they put us in this jail, they take the most important thing for us; our freedom (Alima – female, aged 48)

The qualitative data also revealed some insights into how these refugees' anger and sense of injustice were expressed. For a few, this was expressed outwardly in the form of political action or individual protest and mostly this approach was described as beneficial for wellbeing, insomuch as it was an affirmative demonstration of personal agency and assertion of dignity. By contrast, the experience of anger for other interviewees was more likely to be directed inward at themselves, rather than expressed externally. Some internalised the dominant stereotype of asylum seekers as being 'undeserving,' or interpreted their experiences as god's punishment for previous wrongdoings, while others felt diminished by the treatment they had received by the Australian government. This was more often the case with interviewees with poor language skills, and who lacked education, were unemployed and had diminished family and other supports. This reaction was illustrated by one participant who spent over one in an immigration facility on Nauru. He was interviewed just prior to his voluntary repatriation to Iraq, the prospect of another two years here without his wife and children intolerable. He stated:

I hate myself that I am a refugee because I have seen myself like a miserable person in front of the Australian government...some people they put you down because you are a refugee. (Emir – male, aged 40)

## Discussion

In this study, TPV refugees suffered a higher prevalence of symptoms consistent with clinical depression, higher mean psychological distress and lower sense of wellbeing compared with PHV refugees. Temporary visa status was a significant determinant of psychological distress amongst Iraqi refugees in Melbourne, after controlling for gender, age and marital status. Socioeconomic stressors arising from the denial of core economic and social rights to TPV refugees were significantly more prevalent for TPV versus PHV refugees. However, amongst TPV refugees, psychosocial stressors were more predictive of poor psychological health (i.e. low reported social support and perceived injustice (using anger as a proxy measure). Gender, too, made a significant unique contribution to psychological distress amongst TPV holders. The qualitative data corroborated the survey results. The interview data revealed that female TPV refugees were particularly vulnerable to mental distress, at least in part arising from the emotional burden they shouldered in caring for children and the cultural expectation that they will nurture harmonious relations in the home. The qualitative data also indicated that the psychosocial impact of rights violations in this context predominate over the socioeconomic. While surveyed TPV refugees reported significantly more difficulties in accessing education, English language tuition and welfare, compared with PHV refugees, their narratives were more focused on the pervasive and detrimental impact of the interminable uncertainty about their future, social isolation, anger and sense of injustice.

There are some limitations to the study. In particular, the use of a non-random sampling frame runs this risk of bias in comparing these two groups of refugees. For example, it may be possible that Iraqi refugees with the poorest health were more interested in partaking, perhaps motivated by self-interest. However, prior research suggests it is generally harder to engage such individuals in research projects [44,45] and consequently, it is more likely that the results are an under, rather than an overestimate of the measured health outcomes. It is also notable that the rates of depression found in this study (using a standardised self-report measure) fall within the boundaries of prevalence data previously reported for Iraqi asylum seekers in Australia and Iraqi refugees in the international literature [18,46].

Another source of bias may arise from TPV refugees exaggerating their plight in the hope that this might advance their claims when they applied for permanent protection. While this cannot be completely discounted, the anonymous nature of data collection (i.e. no identifying information was collected) and the clear statement contained in the Information Sheet, which emphasized that the research was independent of DIAC, should have diminished this risk. Additionally, because decisions about ongoing protection are based around risk of future persecution in Iraq, issues relating to living difficulties in Australia, the subject of this research, bear little relevance. The generalisability of the study may also be questioned owing to the sampling methods. In countering this argument, it is noteworthy that the sociodemographic characteristics of the sample were broadly comparable (across age, gender, education and ethnicity) to those of the wider Iraqi refugee community living in the study site according to available data from the DIAC settlement database and other community providers. The extent to which the results are generalisable to other ethnic groups remains uncertain. Finally, transcultural measurement issues are a potential source of measurement error, although considerable effort was invested in the translation and implementation of the survey to guard against this.

Despite these limitations, the study results are broadly confirmatory of a growing body of evidence that Australian asylum policies have been associated with adverse mental health outcomes amongst refugees who have been subject to this regime. Like the two cross-sectional studies preceding this one, temporary visa status was predictive of adverse mental health outcomes amongst refugees. It is noteworthy; however, that length of time in detention was not a significant contributor in this current study, as it was in the study by Steel and colleagues [18]. This may be related to the fact that our study included few participants who had been in detention for a notably prolonged period (e.g. no one over 12 months), which may be more predictive of mental health impact in the long-term (i.e. several years after release). In a similar comparative cross-sectional study with Persian-speaking refugees, it was not length of time in detention but 'stressful' experiences in detention (e.g. exposure to acts of violence, not receiving adequate medical attention etc.) that contributed to PTSD (but not depression) [47]. This suggests that more research may be required to elucidate further the association of length of time in detention and long-term mental illness in this population.

While this study is valuable in adding confirmatory evidence of the adverse health impact of temporary protection of refugees, the originality of this research, is that it extends empirical knowledge about the health impact of human rights violations and the pathways through which rights violations are embodied as poor psychological health outcomes; evidence which has to date been scant. Notably, the results underscore the importance of psychosocial determinants of health, such as social support and sense of injustice, and access to other underlying determinants, such as food and accommodation. That is, the key determinants of the health and wellbeing of TPV refugees cannot be narrowly defined in relation to individual access to health care, but rather are associated with the psychosocial and socioeconomic ramifications of the denial of their human rights. This supports the work of Burris and colleagues [5], who state that "law may be an enormously important pathway along which social structure becomes health destiny in individual lives."

Perhaps most important, is the finding that policies that violate human rights are associated with adverse health outcomes. This link between policy, rights violations and health outcomes offers a framework for addressing the impact of socio-political structures on health. The message for governments is that creating the social conditions for realizing health requires, among other things, that human rights of individuals and collectives be upheld [6,48]. While it is true that many States continue to be remiss in their legal and moral obligations to uphold human rights, there are notable exceptions, like South Africa (with a legally enforceable bill of rights), who have demonstrated that respect for rights can result in improved health outcomes [49].

## Conclusion

Human rights matter for health. In light of this and other corroborative research the Australian federal government needs to be accountable, not only for its legal obligations to uphold human rights, but also for the adverse health consequences arising from policies that violate those rights. To this end, recent Australian migration policy changes to abolish the temporary protection regime and reform immigration detention practices are welcome developments.

Detention of asylum seekers and temporary protection are already used to varying degrees in the US and across some countries in the European Union (EU). Currently in the EU, there are moves towards instituting such practices as standard practice [50]. This study should give pause to States wishing to follow Australia's example.

## Competing interests

The authors declare that they have no competing interests.

## Authors' contributions

VJ developed the study protocol, designed the analysis plan, collected and interpreted the data, and wrote the paper. She will act as guarantor for the paper. PA, KM and MM contributed to the development of the protocol, analysis plan and data interpretation and provided field supervision for the study. They also provided critical feedback on the draft of the paper. All authors read and approved the final manuscript.

## Pre-publication history

The pre-publication history for this paper can be accessed here:

http://www.biomedcentral.com/1472-698X/9/1/prepub
